# Role of proline and pyrroline-5-carboxylate metabolism in plant defense against invading pathogens

**DOI:** 10.3389/fpls.2015.00503

**Published:** 2015-07-06

**Authors:** Aarzoo Qamar, Kirankumar S. Mysore, Muthappa Senthil-Kumar

**Affiliations:** ^1^National Institute of Plant Genome ResearchNew Delhi, India; ^2^Plant Biology Division, The Samuel Roberts Noble Foundation, ArdmoreOK, USA

**Keywords:** P5C, proline, ROS, oxidative burst, hypersensitive response, plant defense, non-host resistance

## Abstract

Pyrroline-5-carboxylate (P5C) is an intermediate product of both proline biosynthesis and catabolism. Recent evidences indicate that proline-P5C metabolism is tightly regulated in plants, especially during pathogen infection and abiotic stress. However, role of P5C and its metabolism in plants has not yet been fully understood. Studies indicate that P5C synthesized in mitochondria has a role in both resistance (*R*)-gene-mediated and non-host resistance against invading pathogens. Proline dehydrogenase and delta-ornithine amino transferase-encoding genes, both involved in P5C synthesis in mitochondria are implicated in defense response of *Nicotiana benthamiana* and *Arabidopsis thaliana* against bacterial pathogens. Such defense response is proposed to involve salicylic acid-dependent pathway, reactive oxygen species (ROS) and hypersensitive response (HR)-associated cell death. Recently HR, a form of programmed cell death (PCD), has been proposed to be induced by changes in mitochondrial P5C synthesis or the increase in P5C levels *per se* in plants inoculated with either a host pathogen carrying suitable avirulent (*Avr*) gene or a non-host pathogen. Consistently, *A. thaliana* mutant plants deficient in P5C catabolism showed HR like cell death when grown in external P5C or proline supplemented medium. Similarly, yeast and plant cells under oxidative stress were shown to increase ROS production and PCD due to increase in P5C levels. Similar mechanism has also been reported as one of the triggers for apoptosis in mammalian cells. This review critically analyzes results from various studies and enumerates the pathways for regulation of P5C levels in the plant cell, especially in mitochondria, during pathogen infection. Further, mechanisms regulating P5C- mediated defense responses, namely HR are outlined. This review also provides new insights into the differential role of proline-P5C metabolism in plants exposed to pathogen infection.

## Introduction

Plant defense against invading pathogen involves complex responses that culminate either in plant susceptibility or resistance. Virulent pathogen colonizes on host plant by surpassing the plant resistance mechanism. Most virulent pathogens have capability to manipulate host gene expression patterns to directly benefit their fitness and cause susceptibility of host plant. However, avirulent pathogen infection provokes *R*-gene mediated resistance owing to recognition of avirulent factor by resistance protein (R-protein). This recognition leads to hypersensitive response (HR)-cell death or other defense responses. Further, a form of defense called non-host resistance is responsible for conferring immunity to a plant species against all races of a potential pathogen (Senthil-Kumar and Mysore, 2013). Non-host resistance is classified into type I and type II resistance (Mysore and Ryu, 2004). Type I resistance does not produce any visible symptom but type II resistance shows HR- cell death as shown in *R*-gene mediated resistance (Senthil-Kumar and Mysore, 2013; see Supplementary Table S1 for terminologies).

These plant defense responses can be systematically categorized in two tiers (Jones and Dangl, 2006) namely, pathogen-associated molecular patterns (PAMPs) triggered immunity (PTI) and effector triggered immunity (ETI). The first tier of immunity, PTI involves perception of pathogen due to PAMPs by plant receptors called pattern recognition receptors (PRRs; Bigeard et al., 2015). This recognition leads to defense response in plant. Some pathogens surpass this tier and are not recognized by plant. Such pathogens release effectors in to the cell to suppress plant defense and acquire nutrition. Then the plant executes second tier of immunity, ETI by which plant recognizes effectors of pathogen by some resistance proteins (R-protein) and prevent pathogen infection (Jones and Dangl, 2006; see Supplementary Table S1 for terminologies). In the past a number of studies had been carried out to unravel the complex resistance mechanisms and susceptibility contributing factors in plants. Recent studies proposed a new line of thinking to explain a part of defense response through proline and 1-pyrroline-5-carboxylic acid (P5C) metabolism.

Proline is a multi-functional imino acid which confers tolerance to plants against abiotic stresses (Hare and Cress, 1997; Lehmann et al., 2010; Szabados and Savoure, 2010) and has been correlated to plant defense against pathogens (Fabro et al., 2004; Cecchini et al., 2011; Senthil-Kumar and Mysore, 2012). Plant accumulates proline by increasing its synthesis and reducing catabolism under abiotic stresses (Kishor et al., 2005; Verbruggen and Hermans, 2008; Supplementary File S1). Proline content was also increased during plant defense against pathogen in *Arabidopsis thaliana* (Fabro et al., 2004; Verslues and Sharma, 2010). However, proline *per se* has not been shown to play a role in defense against pathogen infection. Recent studies have shown that proline catabolism is enhanced during early stages of plant defense against invading pathogens (Cecchini et al., 2011). Based on the evidences from recent studies (Hellmann et al., 2000; Hu et al., 2007; Nishimura et al., 2012; Lee et al., 2013), we speculate that P5C, an intermediate imino acid in proline metabolism, plays important role in plant defense. So far, the role of P5C in plant defense against pathogens is not compiled and discussed in the literature. This review focuses on the role of P5C and its metabolism in plant–pathogen relations and attempts to infuse new thoughts in attributing relevance of P5C metabolism in plants under pathogen infection.

## P5C and Its Metabolism

**FIGURE 1:**
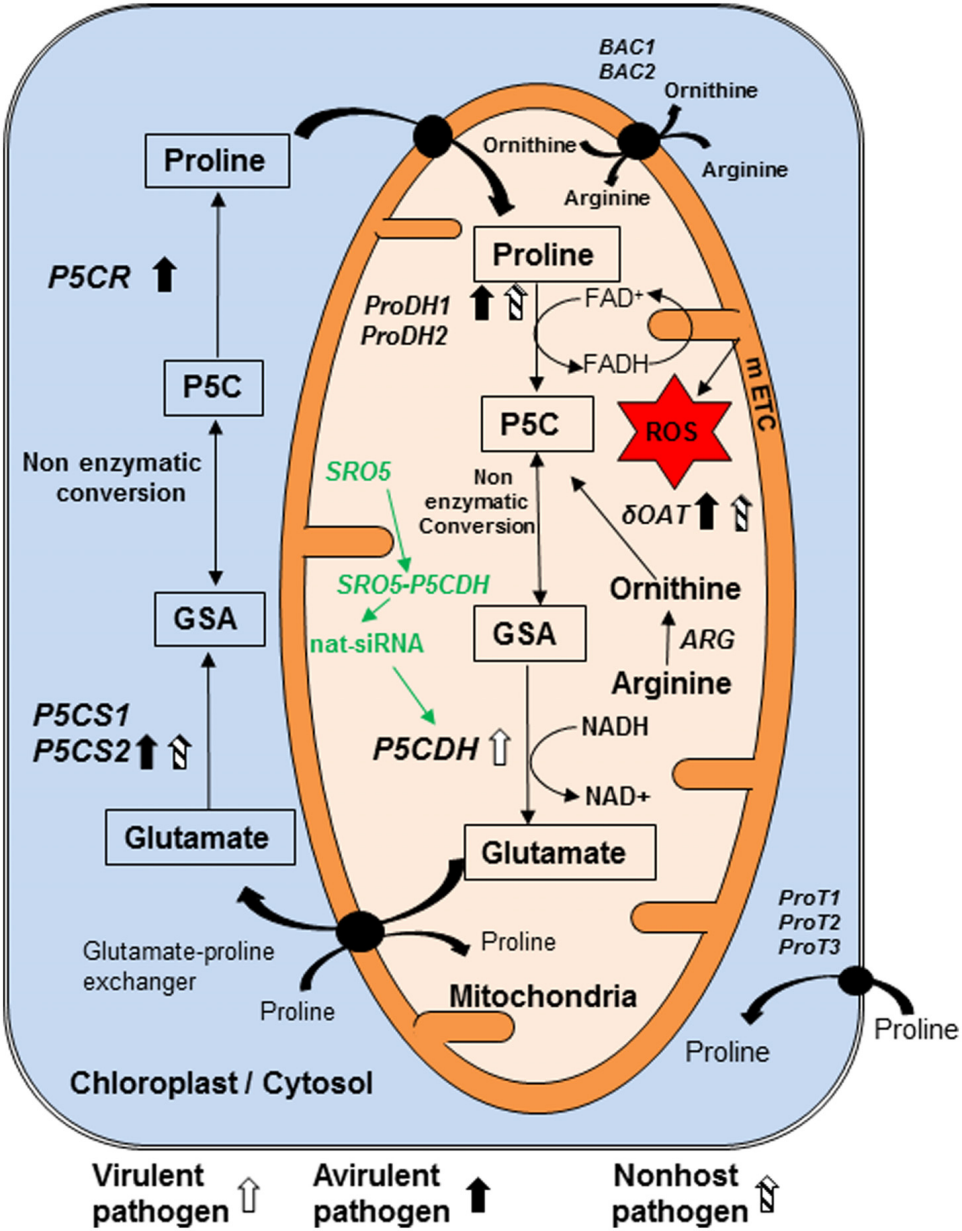
**Model showing genes and pathways possibly involved in synthesis and catabolism of P5C in plant cell and their regulation in response to pathogen infection.** Pyrroline 5-carboxylate (P5C) is the intermediate product of both biosynthesis and catabolism of proline. It is synthesized in mitochondria during catabolism of proline by enzyme proline dehydrogenase (ProDH1/2). We speculate that like their counterparts from bacteria and yeast, this enzyme reduces FAD+ to FADH and increases electron flow in mitochondrial electron transport chain (mETC). Arginine is converted into ornithine by arginase (ARG) enzyme. Another enzyme delta-ornithine amino transferase (δOAT) convert ornithine to P5C in mitochondria. P5C is catabolized by pyrroline 5-carboxylate dehydrogenase (P5CDH) in mitochondria into glutamate. In addition, P5C is synthesized in cytosol and chloroplast, from glutamate by pyrroline 5-carboxylate synthase 1 and 2 (P5CS1, P5CS2) and converted to proline by pyrroline 5-carboxylate reductase (P5CR). P5C and glutamate semi aldehyde (GSA) are non-enzymatically inter-convertible forms. Virulent pathogen infection in plants increases transcript accumulation of *ProDH1*. Avirulent pathogen infection increases transcript accumulation of *P5CS2* and *ProDH1*. Non-host pathogen infection increases transcript accumulation of *δOAT* as well as *ProDH1* and *P5CS2*. Three transporters located on plasma membrane are ProT1, ProT2 and ProT3. Arginine is imported into mitochondria directly and/or in exchange of ornithine by basic amino acid career (BAC1) and BAC2. *P5CDH* gene is down regulated post transcriptionally by natural siRNAs from similar to RCD one-5 (*SRO5*) genes. No direct evidence available for this pathway shown in green color during plant–pathogen interaction and this information is speculated based on evidence available under salt stress. Compounds shown in rectangle are important component of metabolism of P5C and line arrows indicates the direction of synthesis. Curved arrows show the transport of compounds. Transporters shown in circle are present on membrane. Block thick arrows show upregulation of genes, in which white arrow indicates virulent pathogen, dark arrows represent avirulent pathogen and striped arrows indicate non-host pathogen. ROS, reactive oxygen species. This model is predominantly based on information from *Arabidopsis thaliana* literature (Ayliffe et al., 2002; Borsani et al., 2005; Miller et al., 2009; Verslues and Sharma, 2010; Cecchini et al., 2011; Senthil-Kumar and Mysore, 2012).

P5C, an N-substituted imino acid containing imino and carboxyl functional groups (IUPAC, 1997), is an intermediate not only in proline biosynthesis but also in its catabolism (**Figure 1**; Supplementary Table S2). P5C is synthesized from glutamate by pyrroline-5-carboxylate synthase (P5CS; Hu et al., 1992) and then converted to proline by pyrroline-5-carboxylate reductase (P5CR; Szoke et al., 1992; Hare and Cress, 1997) in cytosol and plastids. Proline is transported into mitochondria by membrane located transporters for its catabolism. Proline dehydrogenase (ProDH) catalyzes conversion of proline to P5C, which is then converted to glutamate by pyrroline-5-carboxylate dehydrogenase (P5CDH) in mitochondria (Elthon and Stewart, 1981; Hare and Cress, 1997). In addition to proline catabolism by ProDH (Boggess et al., 1978; Kiyosue et al., 1996), catabolism of arginine to ornithine by arginase (ARG; Goldraij and Polacco, 2000) and later transamination of ornithine by delta-ornithine amino transferase (δOAT) also synthesizes P5C (Delauney et al., 1993; Roosens et al., 1998; Sekhar et al., 2007; Funck et al., 2008; Stránská et al, 2008; Supplementary Table S2; **Figure 1**). P5C remains in rapid equilibrium with glutamate semi-aldehyde (GSA; Vogel and Davis, 1952). This equilibrium is pH dependent and P5C form is favored over GSA at physiological pH of around 7.0 (Lewis et al., 1993; Bearne and Wolfenden, 1995).

## Mitochondrial ROS Accumulation and Cell Death

In plant cells, mitochondria is one of the major sites for the production of reactive oxygen species (ROS) namely superoxide radicals and hydroxyl radicals (Lam et al., 2001; Moller, 2001; Bailey-Serres and Mittler, 2006; Sharma et al., 2012). Electrons are derived from metabolic reducing equivalents [NAD (P) H_2_ and FAD (P) H_2_] and fed into mitochondrial electron transport chain (mETC) through complex I (NADH-ubiquinone oxidoreductase) and/or II (succinate-coenzyme Q reductase) and later transferred to oxygen via terminal oxidase to produce water (Moller, 2001; Arora et al., 2002; Turrens, 2003; Murphy, 2009; Schertl and Braun, 2014). But these carriers can also pass electron directly to oxygen to form superoxide which later produces other forms of ROS and this contributes to cell death (Fredlund, 1996; Lam et al., 2001). A localized cell death known as HR that prevents progression of pathogen infection is an important component of plant defense (Alvarez et al., 1998; Lam et al., 2001). Proline catabolism pathway mediated by ProDH enzyme in mitochondria is one of the sources of mitochondrial ROS during avirulent pathogen infection in *A. thaliana* (Cecchini et al., 2011) and the ROS produced at the infection site leads to oxidative burst thereby initiates HR (Lamb and Dixon, 1997).

There are two schools of thoughts regarding ROS production by P5C metabolism in mitochondria. First one emphasizes the involvement of proline-P5C cycle (Miller et al., 2009) and the extended pathway involving proline-P5C-glutamate in ROS generation. According to proline-P5C cycle, P5C synthesized due to proline catabolism in mitochondria is likely to be transported through an unknown transporter into the cytosol where P5CR enzyme converts it into proline. The proline thus formed is transported back to mitochondria where it consistently acts as a substrate for ProDH enzyme (Miller et al., 2009). In some cases, pathogen infection increased the *ProDH* transcript level and enhanced proline catabolism triggered proline-P5C-proline cycle between mitochondria and cytosol (Cecchini et al., 2011; Monteoliva et al., 2014). This cycle can increase the transfer of reducing equivalents to mitochondria and alter NADP^+^/NADPH ratio in the cytosol (Rejeb et al., 2014). This condition potentially impacts the redox sensitive pathways such as defense-associated oxidative pentose phosphate pathway and eventually produces ROS (Hare and Cress, 1997). ProDH is located in inner membrane of mitochondria toward matrix where it oxidizes proline into P5C and increases electron flow leading to the production of another batch of ROS (Tanner, 2008; Wanduragala et al., 2010; Cecchini et al., 2011; Supplementary Figure S2). In *Trypanosoma cruzi* parasite, *TcProDH* mutation resulted in resistance to oxidative imbalances and ProDH enzyme activity was implicated in ROS production in this study (Paes et al., 2013). However, occurrence of proline-P5C cycle is not conclusively proven. Presence of a P5C transporter at the mitochondrial membrane has only been suggested (Miller et al., 2009) but not yet identified. Similarly the role of genes encoding the mitochondrial transporters that are needed for proline cycle are not well studied. Apart from proline-P5C cycle described above, cyclic metabolisms of proline also occur by other pathways. For example, the extended pathway involving glutamate formation in mitochondria and its cycling back to cytosol for proline synthesis can also be a source of ROS production. However, till date this pathway has not been studied under plant–pathogen interaction.

The second school of thought suggests the role for P5C *per se* in ROS production. Exogenous application of P5C led to apoptosis in human tumor cells through oxidative burst (Maxwell and Davis, 2000). In yeast, overexpression of proline utilization (*PUT1*) gene, which mediates the conversion of proline to P5C, induced cell death (Chen et al., 2006). The *put2* (*PUT2* gene mediates the conversion of P5C to glutamate) mutant grown in proline supplemented minimal medium inhibited yeast growth due to higher ROS production (Deuschle et al., 2001). Similarly, *put2* mutant of a fungal pathogen (*Cryptococcus neoformans*) generated high amount of mitochondrial superoxide radicals and showed cell death in proline supplemented medium (Lee et al., 2013). In addition, overexpression of *ProDH* induced P5C dependent mitochondria-mediated apoptosis in colorectal cancer cells (Hu et al., 2007). Proline-induced cell death in yeast was also shown to be due to increased P5C levels in mitochondria (Nomura and Takagi, 2004). Nishimura et al. (2012) reported that accumulation of P5C is responsible for mitochondrial ROS production and hence cell death in yeast. Similarly, *A. thaliana p5cdh* mutant, which cannot catabolize P5C, and *ProDH* overexpression lines accumulated P5C in mitochondria and were hypersensitive to exogenous application of proline, ornithine, and arginine (Deuschle et al., 2004). The enhanced catabolism of proline due to increased expression of *AtProDH* in the *p5cdh* mutant plants led to P5C accumulation with concomitant cell death (Deuschle et al., 2004). Also, *AtP5CDH* overexpressed plants did not show cell death but wild-type *A. thaliana* plants grown on MS medium supplemented with P5C showed hypersensitivity (Miller et al., 2009). These evidences suggest the role for P5C *per se* in ROS generation and eventually cell death (Supplementary Figure S2). P5C spray on wild-type *A. thaliana* leaves resulted in HR like cell death with concomitant callose accumulation (Deuschle et al., 2004). Similarly, proline hypersensitivity in wild-type *A. thaliana* leaves manifested as necrosis and browning symptoms were attributed to increased cellular P5C (Hellmann et al., 2000). Taken together, these studies indicate that proline-P5C cycle and/or P5C *per se* can contribute to HR to confine pathogen growth in plants.

## Role of P5C Metabolism During Plant-Avirulent Pathogen Interactions

HR occurs during interaction of plant with either a host pathogen carrying avirulent (*Avr*) gene, for which plant contains corresponding *R* gene, or a non-host pathogen (Ausubel et al., 1995; Boyes et al., 1998; Mysore and Ryu, 2004; Senthil-Kumar and Mysore, 2013). Role of P5C metabolism under both type of resistance are discussed in this section. *Pseudomonas syringae* pv *tomato* DC3000 (causal agent of bacterial spec disease in tomato) carrying *AvrRpm1* or *AvrRpt2* gene produces AvrRPM1 or AvrRPT2 effector protein, respectively. These are recognized by resistance to *P. syringae maculicola* 1 (RPM1) or resistance to *P. syringae* 2 (RPS2) protein, respectively, in host plant through RPM interacting protein (RIN4) (Mackey et al., 2002; Axtell and Staskawicz, 2003). This recognition leads to changes in proline-P5C metabolism (Cecchini et al., 2011; Monteoliva et al., 2014) and up regulation of various defense genes and ROS production culminating in HR-cell death (Torres et al., 2006). External proline application, that can provoke P5C accumulation induces pathogenesis related 1 (*PR1*) gene expression in *A. thaliana* (Chen et al., 2011). These recent studies highlight the role of P5C and its metabolism in inducing defense responses, including HR during plant–pathogen interactions.

Fabro et al. (2004) reported the accumulation of *AtP5CS2* transcripts and increased proline content prior to HR in *A. thaliana* plants infected with avirulent pathogens (*P. syringae* pv *tomato* DC3000 *AvrRpt2* and *P. syringae* pv *tomato* DC3000 *AvrRpm1*). Cecchini et al. (2011) reported upregulation of *AtProDH1* and *AtProDH2* genes in *A. thaliana* at the inoculation site of avirulent pathogen (*P. syringae* pv *tomato* DC3000 *AvrRpm1*). In addition, avirulent pathogen inoculation led to increased ProDH protein content and enzyme activity and was suggested to be responsible for oxidative burst and HR (Cecchini et al., 2011). Consistently, *AtProDH* silenced *A. thaliana* plants inoculated with avirulent pathogen compromised ROS production (Cecchini et al., 2011). *AtProDH* gene transcript expression was modulated by exogenous application of salicylic acid (SA, Cecchini et al., 2011). Authors in this study proposed that SA-mediated signaling plays a role in the induction of *AtProDH1*, but not *AtProDH2*, expression likely through non-expressor of *PR* genes 1 (*NPR1*) and SA induction-deficient 2 (*SID2*) during early stages of avirulent pathogen infection in *A. thaliana*. Proline was shown to induce SA via non-responsive to disease resistance 1 (NDR1)-dependent pathway (Vlot et al., 2009; Chen et al., 2011). In green bean (*Phaseolus vulgaris*), *Rhizoctonia solani* (causal agent of seed rot and damping off in bean) infection induced proline accumulation in SA-dependent manner (Ayoubi and Soleimani, 2014). Taken together, these studies implicate a role for *AtP5CS2* and *AtProDH1* genes in regulating the proline and P5C under Avr-R interaction. The defense responses involving these genes are likely part of ETI and involve SA.

Further, *AtP5CDH* gene transcription was downregulated in *A. thaliana* upon inoculation with *P. syringae* pv. *tomato* DC3000 *AvrRpm1*. Consistently in *p5cdh* mutant of *A. thaliana*, slight increase in P5C level was observed in the HR region upon inoculation by *P. syringae* pv. *tomato* DC3000 *AvrRpm1* (Monteoliva et al., 2014). This scenario facilitate decrease in degradation of P5C and hence results in high accumulation of P5C (Supplementary Figures S1 and S2). Notably, slight increase in concentration of P5C due to induction of P5C biosynthesis by avirulent pathogen inoculation had been shown to provoke HR (Hellmann et al., 2000; Nishimura et al., 2012). It is likely that *AtP5CDH*-mediated P5C regulation is also a part of ETI.

Similar to avirulent pathogens, non-host pathogen infection also involves HR and other defenses likely mediated by P5C metabolism (Senthil-Kumar and Mysore, 2012, 2013). Recently, virus-induced gene silencing (VIGS)-based forward genetics screen identified the role for *ProDH1* and *δOAT* in non-host resistance (Senthil-Kumar and Mysore, 2012). Silencing of *NbδOAT* and *NbProDH1* genes compromised non-host resistance against *P. syringae* pv. *tomato* T1 in *Nicotiana benthamiana*. Further, *A. thaliana δoat* and *p5cdh* mutant plants lacked oxidative burst and compromised non-host resistance against *P. syringae* pv. *tabaci* (Senthil-Kumar and Mysore, 2012). These mutant plants grown in Murashige and Skoog (MS) medium externally supplemented with P5C showed higher ROS content as compared to those grown in non-P5C medium. Taken together, this study suggested the possible involvement of P5C synthesis step in non-host resistance, possibly as a part of both PTI and ETI.

## Role of P5C Metabolism During Plant-Virulent Pathogen Interaction

**FIGURE 2:**
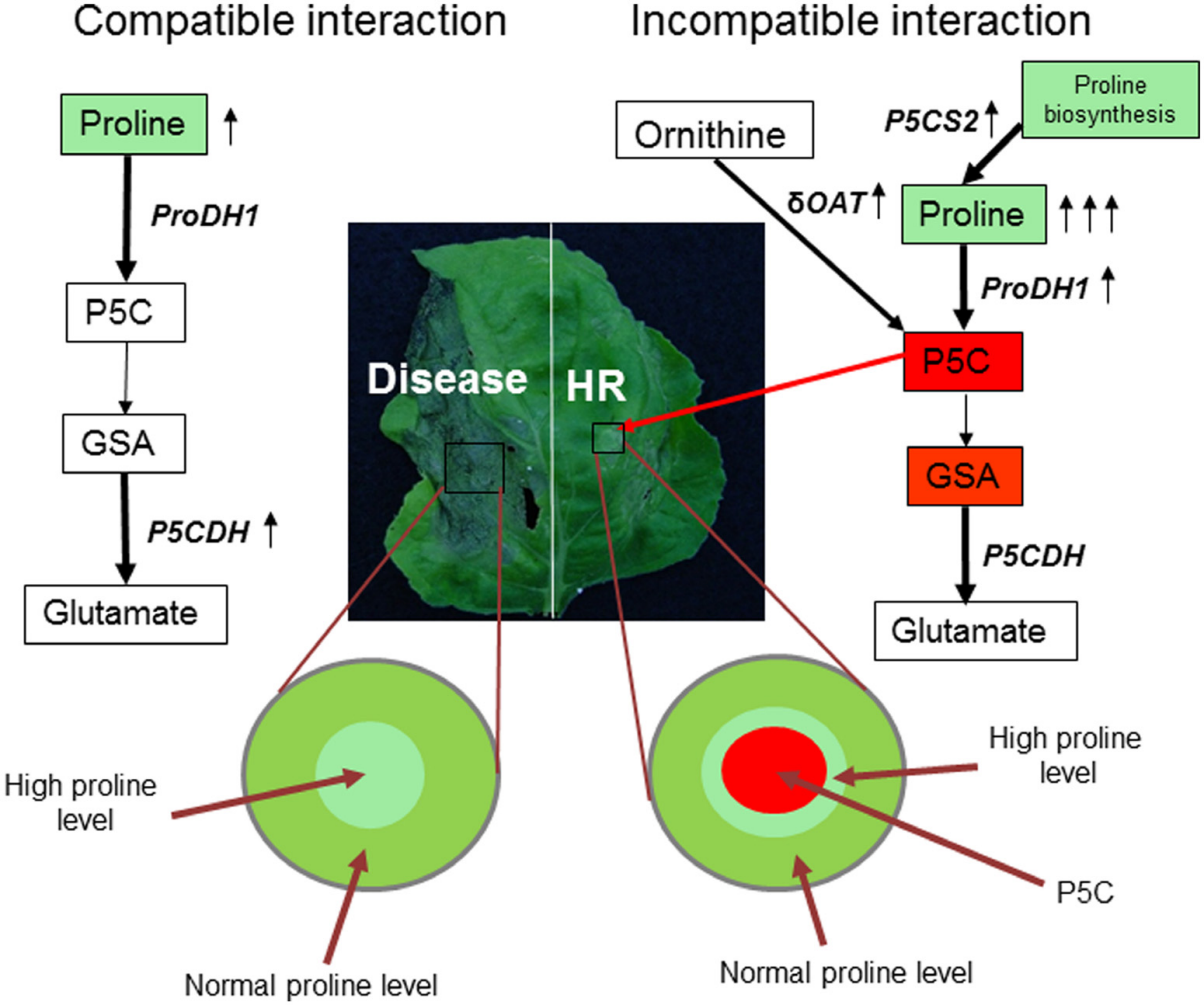
**Model for coordinated regulation of proline-P5C metabolism under pathogen infection.** Virulent pathogen (example as shown here for *Pseudomonas syringae* pv. tabaci in *Nicotiana benthamiana*) infection leads to disease on host plant **(left)**. However, non-host (example as shown here for *P. syringae* pv. tomato T1 in *N. benthamiana*) [and avirulent, not shown in the picture] pathogen infection on plant leads to defense reaction, including HR at infection site **(right)**. Pathways leading to P5C biosynthesis and catabolism in mitochondria are highlighted. Plants increase P5C content or ProDH-mediated step and produce ROS to defend against pathogen by upregulating *ProDH* and *δOAT* gene transcription. In addition, mild increase in proline in the tissues surrounding the HR cell death prevents runaway HR to non-infected cells. However, virulent pathogens decrease P5C levels in mitochondria by upregulating P5CDH-mediated step. Also proline content moderately increases in the infected cells possibly to decrease the rate of disease progression as a part of basal defense response. Proline is known to prevent or delay the cell death. During this regulation, proline synthesis can also play a role in controlling proline levels (shown under resistant reaction). Red rectangle box depicts pro cell death compound; green rectangle depicts compound that delays cell death; arrow depicts upregulation of transcripts.

Flax (*Linum usitatissimum*) plant infected with the *Melampsora lini* (causal agent of flax rust), an obligate biotrophic fungal pathogen, induced the *FIS1* (flax inducible sequence 1) gene at the pathogen infection site (Ayliffe et al., 2002). Interestingly, *FIS1* gene showed 73% nucleotide homology with the *A. thaliana AtP5CDH* gene (Mitchell et al., 2006). Similarly, *N. benthamiana* plants infected with virulent pathogen (*P. syringae* pv. *tabaci*) showed upregulation of *NbP5CDH* gene expression (Senthil-Kumar and Mysore, 2012). It is possible that virulent pathogens enhance the catabolism of P5C by upregulating *AtP5CDH*-mediated step (**Figure 2**; Supplementary Figure S1). In *A. thaliana*, infection by virulent pathogen (*P. syringae* pv. *tomato* DC3000) did not lead to upregulation of *ProDH* gene (Cecchini et al., 2011). Similarly, *N. benthanmiana* plants also did not show upregulation of *NbProDH1* and *NbProDH2* gene expression at *P. syringae* pv. *tabaci* infection site (Senthil-Kumar and Mysore, 2012). This indicates that plants attempt to maintain proline levels in the cells undergoing disease induced cell death. This is consistent with previous studies that indicated the role of proline in reducing cell death in yeast (Chen et al., 2006), fungus (Chen and Dickman, 2005), and plants (Banu et al., 2009). Proline provides protection against H_2_O_2_-induced cell death and apoptosis in mammalian cell culture (Krishnan et al., 2008). Taken together, these studies demonstrated that proline, not P5C, is likely to play a role in regulating disease-induced cell death (**Figure 2**). In addition to bacterial and fungal pathogens, virus infection was shown to influence proline-P5C metabolism. For example, rice plants infected with *Brome mosaic virus* (BMV) or *Rice tungro virus* led to proline accumulation (Mohanty and Sridhar, 1982; Xu et al., 2008). However, the positive role of proline in cell death regulation is not examined in these studies.

## Coordinated Regulation of Proline-P5C Metabolism During Plant Defense

High proline accumulation in non-stressed plants has been shown to negatively impact plant growth and also cause cell death (Hellmann et al., 2000; Chen et al., 2011). However, when these plants are subjected to stress the toxicity due to proline accumulation is reduced. The proline content increases upon pathogen infection (Fabro et al., 2004), but exogenous proline application to non-stressed plants cause increase in its catabolism (Verslues and Sharma, 2010). Proline itself at low concentrations is known to reduce the cell death, but P5C metabolism provokes cell death (Deuschle et al., 2004; Banu et al., 2009). These evidences indicate the complex but fine-tuned regulation of proline-P5C metabolism during pathogen infection (**Figure 2**). It is possible that in virulent pathogen infected cells, proline acts as a positive regulator of cell death as part of basal immune response to decrease the severity of disease development. It is also possible that the un-infected cells surrounding the HR developing cells upon inoculation with avirulent pathogen increase proline levels to prevent run-away cell death (Senthil-Kumar and Mysore, 2012). In contrary, either proline-P5C cycle or P5C accumulation *per se* triggers the defense cell death (HR) upon avirulent pathogen infection. In order to bring a fine-tuned regulation of proline-P5C pathway the plant must regulate the genes involved in defense through a systematic network. We speculate that this network may involve a tight regulation at the promoters. Yet another possibility of post transcriptional regulation could involve micro RNAs (miRNAs). In potato, miRNAs targeting proline metabolism genes were predicted and their role in regulation was verified (Yang et al., 2013). We speculate that proline metabolism genes can be regulated by microRNAs under pathogen attack as well.

Additionally, expression of proline metabolism genes is differentially regulated by drought, salinity, and abscisic acid suggesting that these genes play a specific role in the control of proline synthesis (Supplementary File S1). This indicates the coordinated regulation of proline-P5C metabolism under abiotic stress as well. Considering literature information available on their role under abiotic stresses, we comprehensively illustrated this information together with the regulation under plant–pathogen interaction. Both proline and P5C content under various abiotic stresses changes and regulation of genes involved in different steps of proline catabolism can be correlated to their levels under pathogen stress.

## Concluding Remarks

P5C plays a role in HR against invading pathogen and its levels or metabolism could possibly be manipulated by virulent pathogen. To know the importance of its metabolism in plant defense, it is necessary to understand the regulation of genes involved in its pathway at transcriptional and post transcriptional level. Bioinformatic analysis and experimental validation of promoter region of all proline-P5C metabolism genes will be useful for understanding the regulatory components. In addition, studies to understand post transcriptional modifications, cofactor binding, protein interactors and activity of all proteins involved in the pathway during plant–pathogen interaction are required.

It is still not clear whether the changes in proline-P5C metabolism are primarily regulated by ETI or PTI or both. Systematic studies that will exclusively use PAMPs, effectors and specific triggers for innate immunity is needed to delineate the defense pathway that provoke changes in P5C metabolism. In addition to this, the downstream defense pathway activated by P5C that culminates in HR or other defense responses is also not well understood. Studies using externally supplied purified P5C and genetically manipulated plants with altered P5C levels under a specific defense inducer can provide useful information. Non-host plant–pathogen interaction can be used as model to study both defense involving HR and others. This is because both type-I (HR not involved) and type-II (involving HR) defenses can be tested using the same plant species.

More studies are needed to understand whether P5C content in mitochondria, suppression of its catabolism or its transport plays a role in plant defense. The contribution of cytosolic P5C in pathogen induced plant defense is also not yet studied. In order to understand whether the level of P5C plays any role in triggering HR, it is necessary to accurately estimate the P5C content. However, currently available methods do not provide organelle specific accurate estimation of this compound. For example, colorimetric method of P5C estimation by *O*-amino-benzaldehyde assay is not sensitive enough to estimate P5C content *in vivo*. Hence, development of suitable P5C estimation method is important.

Since proline is well known to play a positive role during drought stress and also during stress recovery, it will be interesting to study the plant’s interaction with virulent and avirulent pathogens under these two conditions. Further, studies on proline pathway gene regulation under the combined drought and pathogen infection will be useful.

## Conflict of Interest Statement

The Review Editor Yasuhiro Ishiga declares that, despite having collaborated and co-authored manuscripts with the authors, Mysore and Senthil-Kumar, the review process was conducted objectively. The authors declare that the research was conducted in the absence of any commercial or financial relationships that could be construed as a potential conflict of interest.
