# Supplementary material for: Role of proline and pyrroline-5-carboxylate metabolism in plant defense against invading pathogens
Source: Front Plant Sci. 2015 Jul 6;6:503. doi: 10.3389/fpls.2015.00503 (PMC4491715; doi:10.3389/fpls.2015.00503)
Supplement: Supplementary file 1 [file Presentation_1.ZIP › presentation 1/Senthil-Kumar_SupplementaryMaterial/Supplementary figure S1.PPTX]

## Slide 1
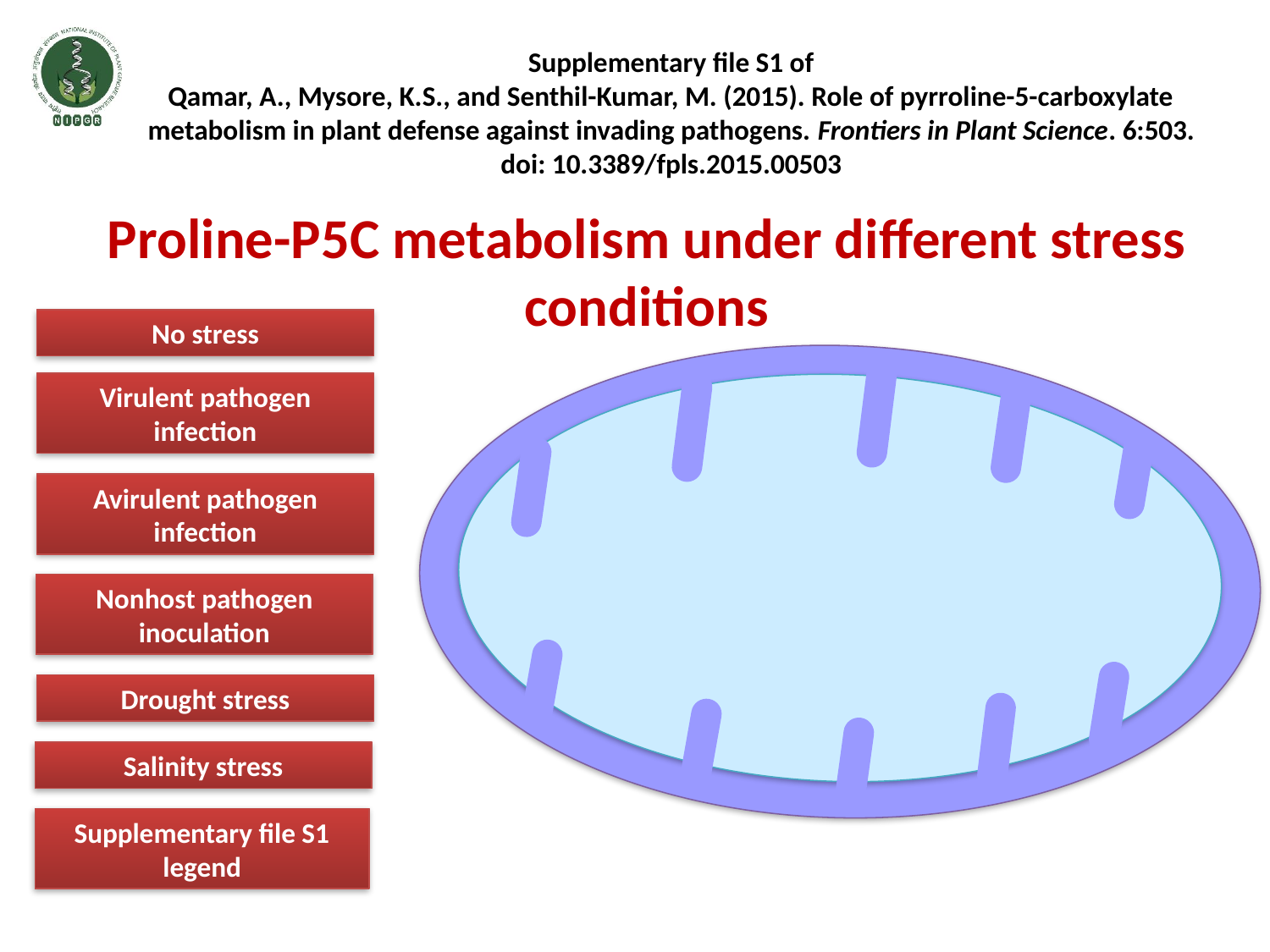

Supplementary file S1 of
Qamar, A., Mysore, K.S., and Senthil-Kumar, M. (2015). Role of pyrroline-5-carboxylate metabolism in plant defense against invading pathogens. Frontiers in Plant Science. 6:503. doi: 10.3389/fpls.2015.00503
Proline-P5C metabolism under different stress conditions
No stress
Virulent pathogen infection
Avirulent pathogen infection
Nonhost pathogen inoculation
Drought stress
Salinity stress
Supplementary file S1 legend

## Slide 2
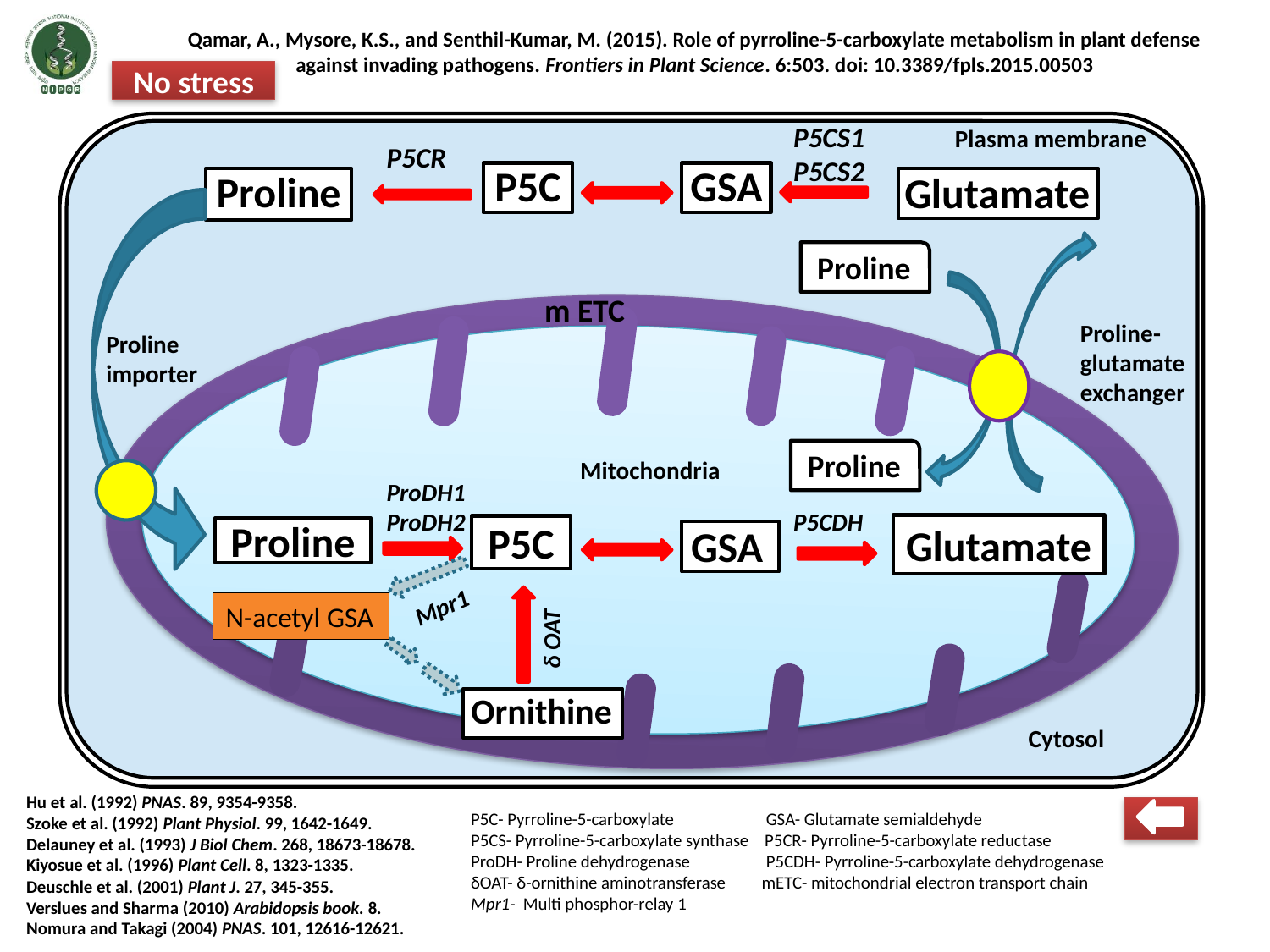

Qamar, A., Mysore, K.S., and Senthil-Kumar, M. (2015). Role of pyrroline-5-carboxylate metabolism in plant defense against invading pathogens. Frontiers in Plant Science. 6:503. doi: 10.3389/fpls.2015.00503
No stress
P5CS1
P5CS2
P5CR
P5C
GSA
Proline
Glutamate
Proline
Proline
ProDH1
ProDH2
P5CDH
GSA
Glutamate
P5C
Proline
δ OAT
Ornithine
Plasma membrane
m ETC
Proline- glutamate exchanger
Proline importer
Mitochondria
Cytosol
Hu et al. (1992) PNAS. 89, 9354-9358.
Szoke et al. (1992) Plant Physiol. 99, 1642-1649.
Delauney et al. (1993) J Biol Chem. 268, 18673-18678.
Kiyosue et al. (1996) Plant Cell. 8, 1323-1335.
Deuschle et al. (2001) Plant J. 27, 345-355.
Verslues and Sharma (2010) Arabidopsis book. 8.
Nomura and Takagi (2004) PNAS. 101, 12616-12621.
P5C- Pyrroline-5-carboxylate GSA- Glutamate semialdehyde
P5CS- Pyrroline-5-carboxylate synthase P5CR- Pyrroline-5-carboxylate reductase
ProDH- Proline dehydrogenase P5CDH- Pyrroline-5-carboxylate dehydrogenase
δOAT- δ-ornithine aminotransferase mETC- mitochondrial electron transport chain
Mpr1- Multi phosphor-relay 1
Mpr1
N-acetyl GSA

## Slide 3
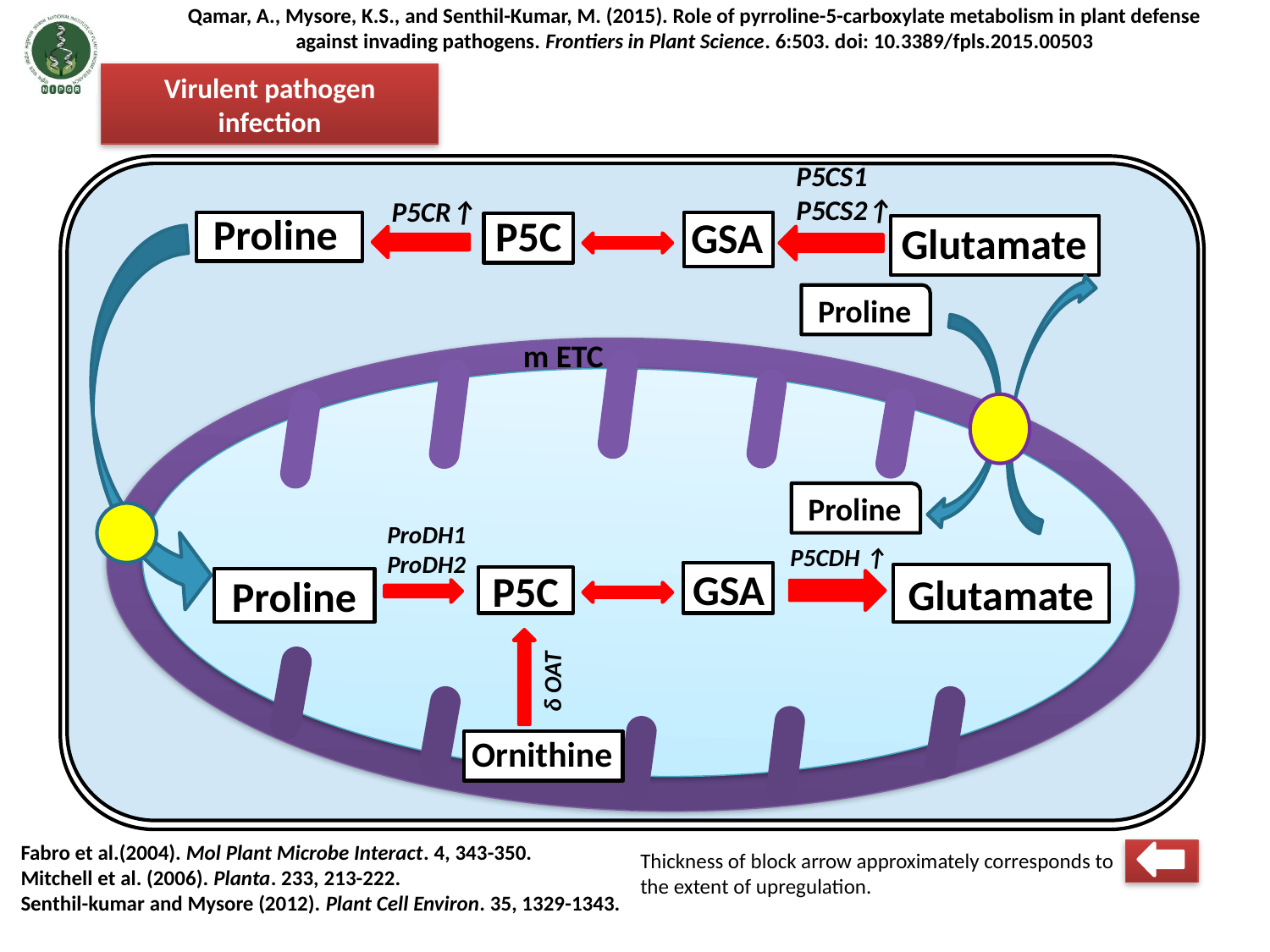

Qamar, A., Mysore, K.S., and Senthil-Kumar, M. (2015). Role of pyrroline-5-carboxylate metabolism in plant defense against invading pathogens. Frontiers in Plant Science. 6:503. doi: 10.3389/fpls.2015.00503
Virulent pathogen infection
P5CS1
P5CS2↑
P5CR↑
Proline
P5C
GSA
Glutamate
Proline
Proline
ProDH1
ProDH2
P5CDH ↑
GSA
Glutamate
P5C
Proline
δ OAT
Ornithine
m ETC
Fabro et al.(2004). Mol Plant Microbe Interact. 4, 343-350.
Mitchell et al. (2006). Planta. 233, 213-222.
Senthil-kumar and Mysore (2012). Plant Cell Environ. 35, 1329-1343.
Thickness of block arrow approximately corresponds to the extent of upregulation.

## Slide 4
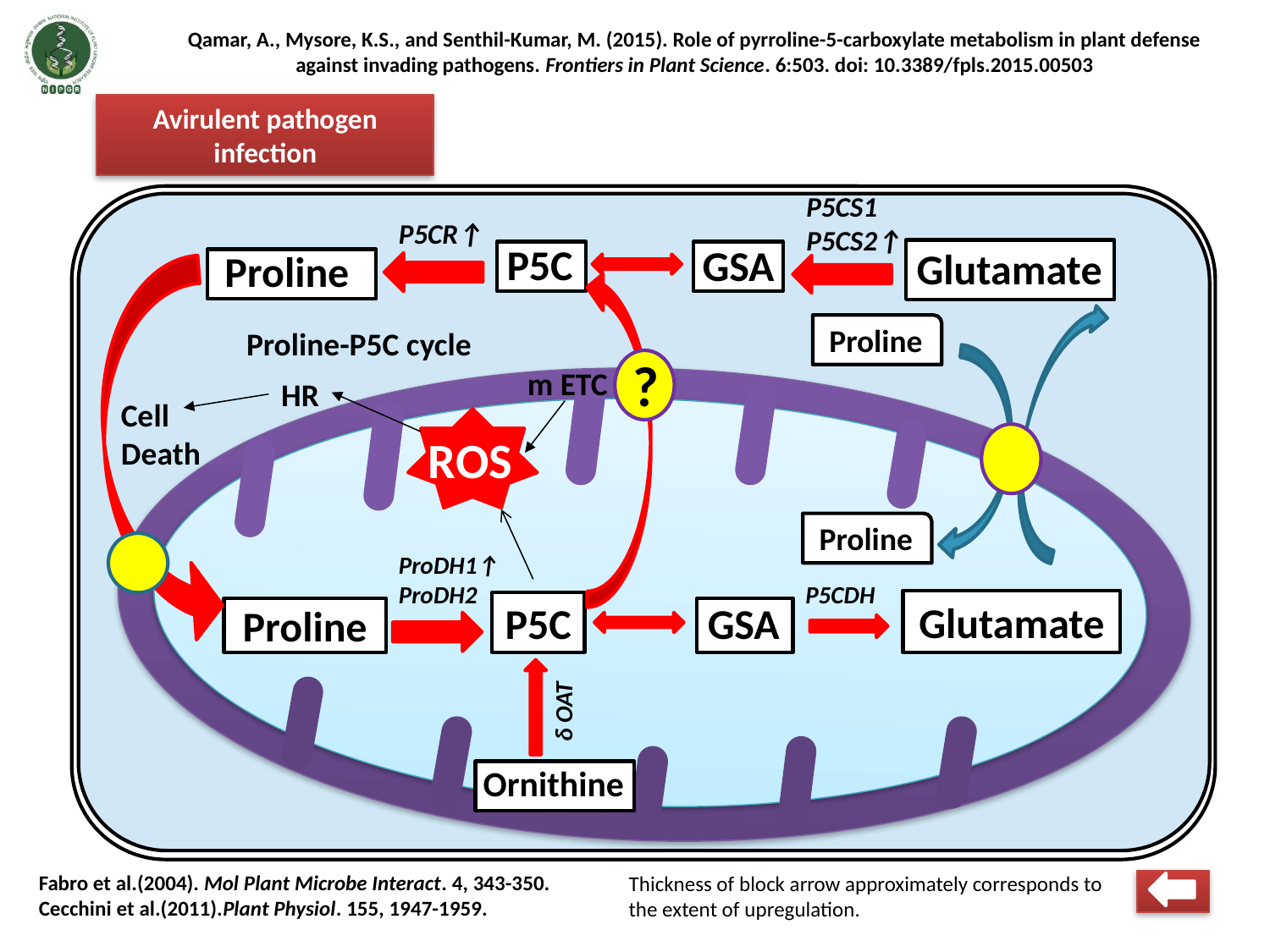

Qamar, A., Mysore, K.S., and Senthil-Kumar, M. (2015). Role of pyrroline-5-carboxylate metabolism in plant defense against invading pathogens. Frontiers in Plant Science. 6:503. doi: 10.3389/fpls.2015.00503
Avirulent pathogen infection
P5CS1
P5CS2↑
P5CR↑
P5C
GSA
Glutamate
Proline
Proline
Proline
ProDH1↑
ProDH2
P5CDH
Glutamate
GSA
P5C
Proline
δ OAT
Ornithine
Proline-P5C cycle
?
m ETC
HR
Cell Death
ROS
Fabro et al.(2004). Mol Plant Microbe Interact. 4, 343-350.
Cecchini et al.(2011).Plant Physiol. 155, 1947-1959.
Thickness of block arrow approximately corresponds to the extent of upregulation.

## Slide 5
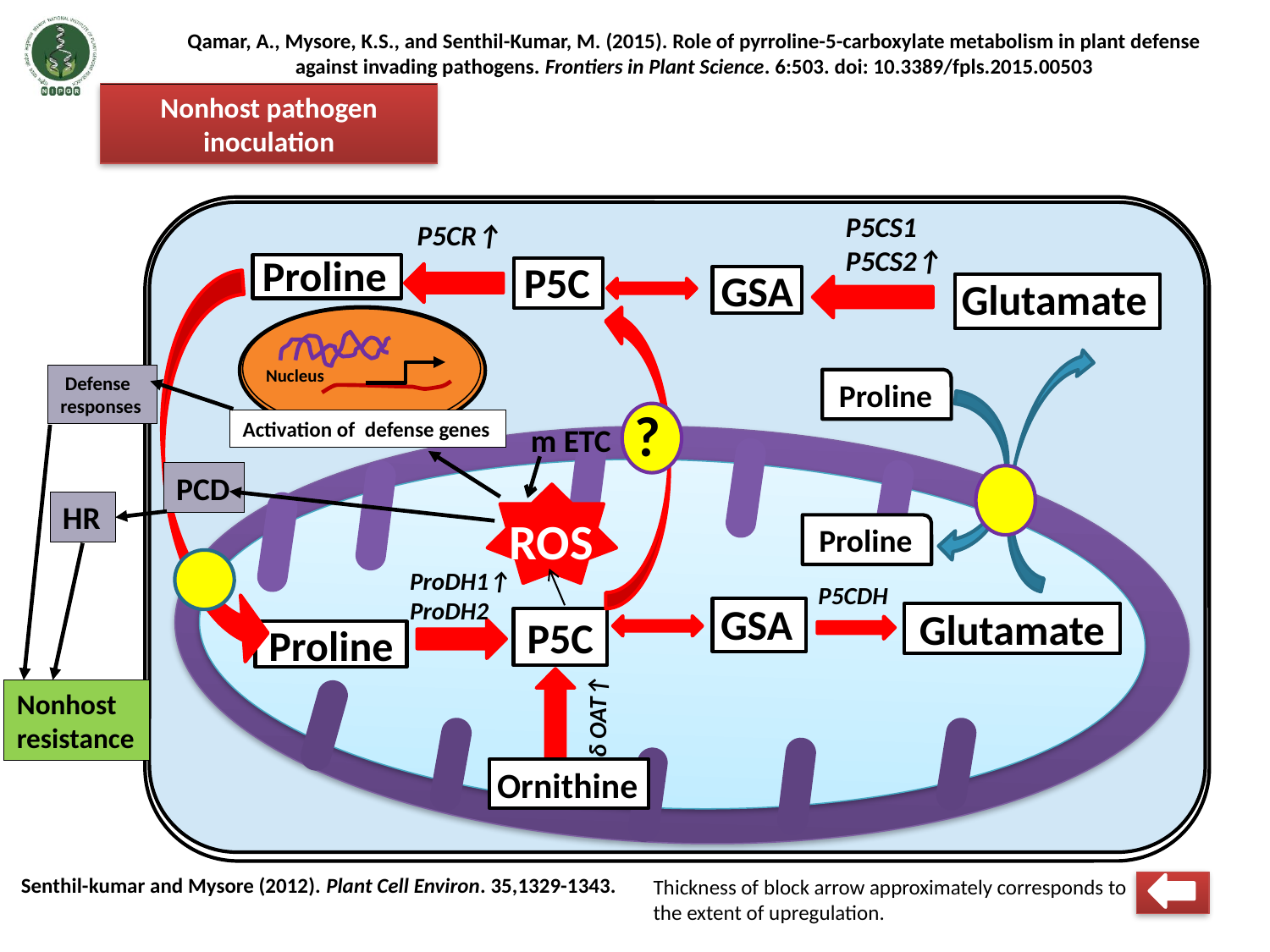

Qamar, A., Mysore, K.S., and Senthil-Kumar, M. (2015). Role of pyrroline-5-carboxylate metabolism in plant defense against invading pathogens. Frontiers in Plant Science. 6:503. doi: 10.3389/fpls.2015.00503
P5CS1
P5CS2↑
P5CR↑
Proline
P5C
GSA
Glutamate
Proline
Proline
ProDH1↑
ProDH2
P5CDH
GSA
Glutamate
P5C
Proline
δ OAT↑
Ornithine
?
m ETC
PCD
ROS
Thickness of block arrow approximately corresponds to the extent of upregulation.
Nonhost pathogen inoculation
 Defense responses
Activation of defense genes
HR
Nonhost resistance
Senthil-kumar and Mysore (2012). Plant Cell Environ. 35,1329-1343.
Nucleus

## Slide 6
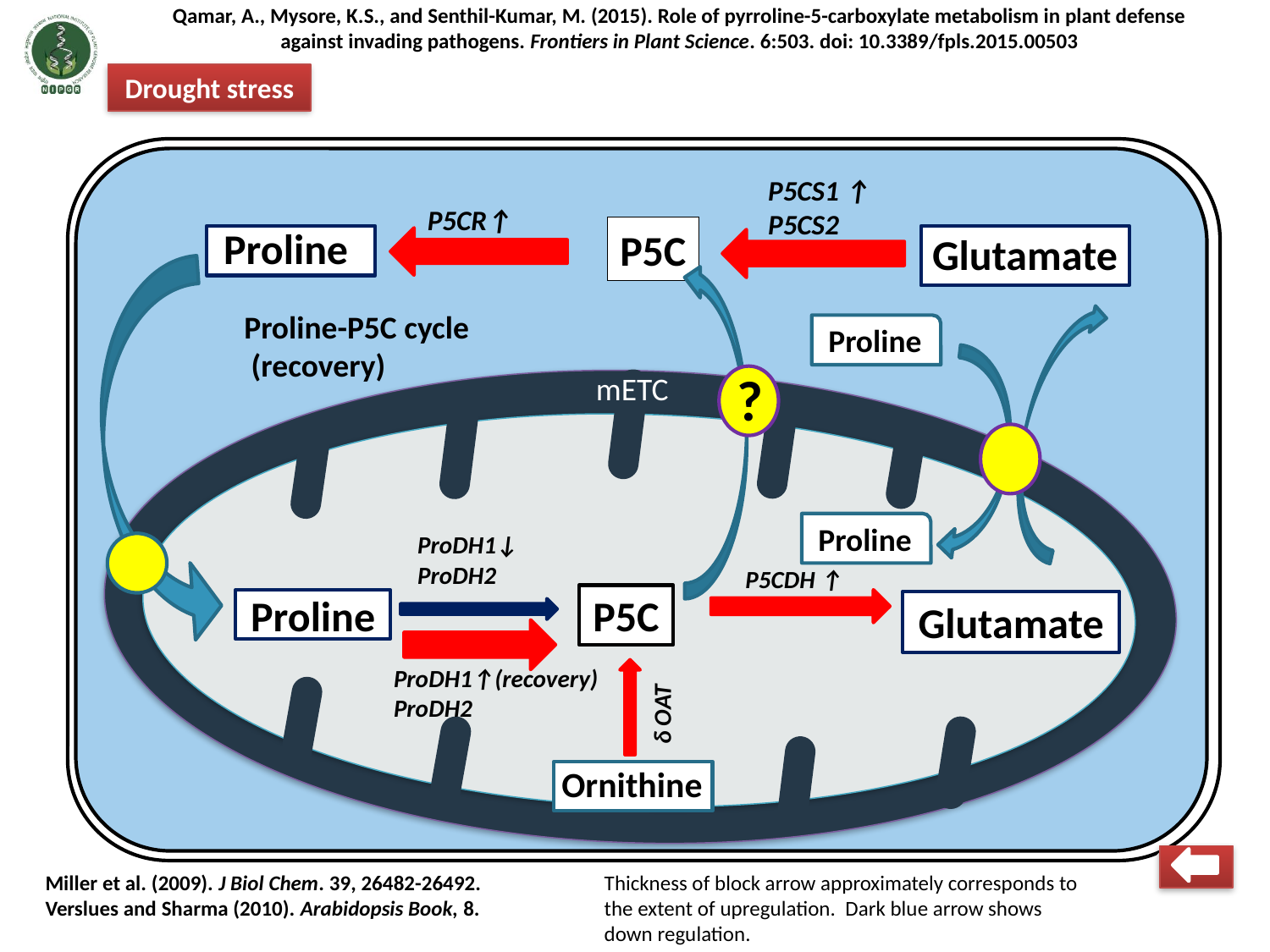

Qamar, A., Mysore, K.S., and Senthil-Kumar, M. (2015). Role of pyrroline-5-carboxylate metabolism in plant defense against invading pathogens. Frontiers in Plant Science. 6:503. doi: 10.3389/fpls.2015.00503
Drought stress
P5CS1 ↑
P5CS2
P5CR↑
Proline
P5C
Glutamate
Proline-P5C cycle (recovery)
Proline
?
mETC
Proline
ProDH1↓
ProDH2
P5CDH ↑
P5C
Proline
Glutamate
δ OAT
Ornithine
Miller et al. (2009). J Biol Chem. 39, 26482-26492.
Verslues and Sharma (2010). Arabidopsis Book, 8.
Thickness of block arrow approximately corresponds to the extent of upregulation. Dark blue arrow shows down regulation.
ProDH1↑(recovery)
ProDH2

## Slide 7
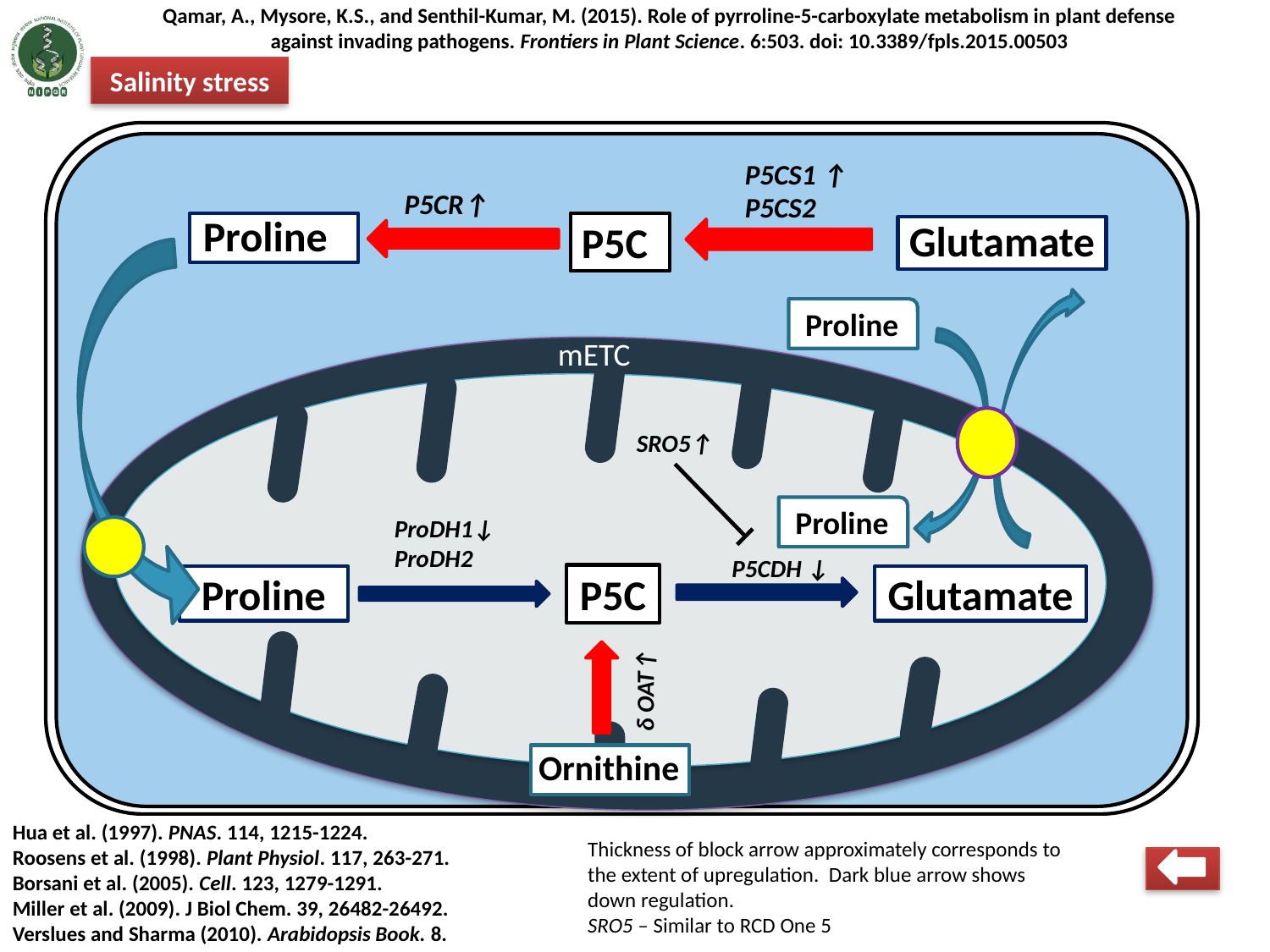

Qamar, A., Mysore, K.S., and Senthil-Kumar, M. (2015). Role of pyrroline-5-carboxylate metabolism in plant defense against invading pathogens. Frontiers in Plant Science. 6:503. doi: 10.3389/fpls.2015.00503
Salinity stress
P5CS1 ↑
P5CS2
P5CR↑
Proline
Glutamate
P5C
Proline
mETC
Proline
ProDH1↓
ProDH2
P5CDH ↓
P5C
Proline
Glutamate
δ OAT↑
Ornithine
Hua et al. (1997). PNAS. 114, 1215-1224.
Roosens et al. (1998). Plant Physiol. 117, 263-271.
Borsani et al. (2005). Cell. 123, 1279-1291.
Miller et al. (2009). J Biol Chem. 39, 26482-26492.
Verslues and Sharma (2010). Arabidopsis Book. 8.
Thickness of block arrow approximately corresponds to the extent of upregulation. Dark blue arrow shows down regulation.
SRO5 – Similar to RCD One 5
SRO5↑

## Slide 8
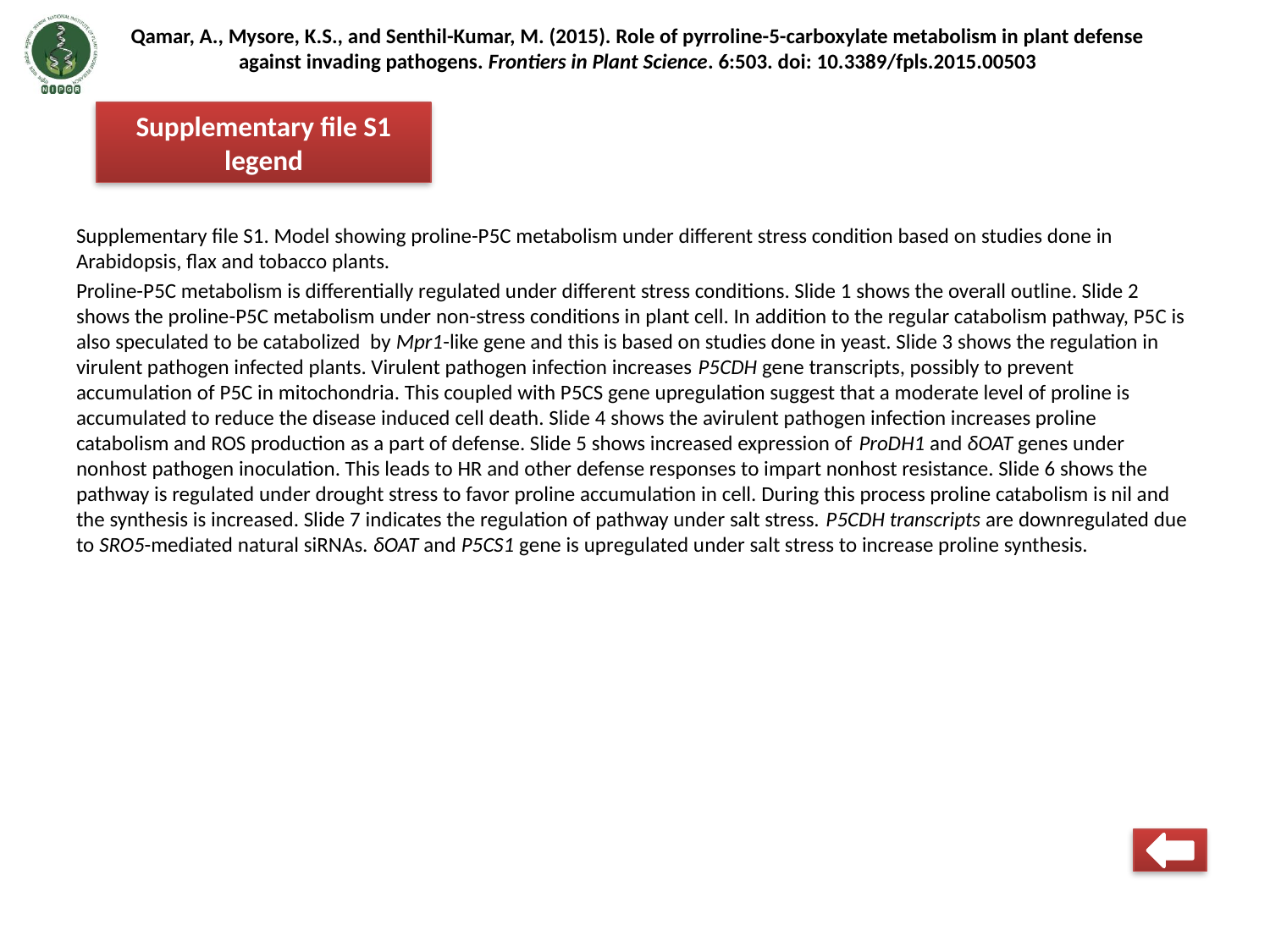

Qamar, A., Mysore, K.S., and Senthil-Kumar, M. (2015). Role of pyrroline-5-carboxylate metabolism in plant defense against invading pathogens. Frontiers in Plant Science. 6:503. doi: 10.3389/fpls.2015.00503
Supplementary file S1 legend
Supplementary file S1. Model showing proline-P5C metabolism under different stress condition based on studies done in Arabidopsis, flax and tobacco plants.
Proline-P5C metabolism is differentially regulated under different stress conditions. Slide 1 shows the overall outline. Slide 2 shows the proline-P5C metabolism under non-stress conditions in plant cell. In addition to the regular catabolism pathway, P5C is also speculated to be catabolized by Mpr1-like gene and this is based on studies done in yeast. Slide 3 shows the regulation in virulent pathogen infected plants. Virulent pathogen infection increases P5CDH gene transcripts, possibly to prevent accumulation of P5C in mitochondria. This coupled with P5CS gene upregulation suggest that a moderate level of proline is accumulated to reduce the disease induced cell death. Slide 4 shows the avirulent pathogen infection increases proline catabolism and ROS production as a part of defense. Slide 5 shows increased expression of ProDH1 and δOAT genes under nonhost pathogen inoculation. This leads to HR and other defense responses to impart nonhost resistance. Slide 6 shows the pathway is regulated under drought stress to favor proline accumulation in cell. During this process proline catabolism is nil and the synthesis is increased. Slide 7 indicates the regulation of pathway under salt stress. P5CDH transcripts are downregulated due to SRO5-mediated natural siRNAs. δOAT and P5CS1 gene is upregulated under salt stress to increase proline synthesis.
