# Supplementary material for: Role of proline and pyrroline-5-carboxylate metabolism in plant defense against invading pathogens
Source: Front Plant Sci. 2015 Jul 6;6:503. doi: 10.3389/fpls.2015.00503 (PMC4491715; doi:10.3389/fpls.2015.00503)
Supplement: Supplementary file 1 [file Presentation_1.ZIP › presentation 1/Senthil-Kumar_SupplementaryMaterial/Supplementary table S1.DOCX]

**Supplementary table S1. Definitions of plant-pathogen interaction related terms used in this manuscript**

| **Sl. No.** | **Terminology** | **Definition** |
| --- | --- | --- |
| 1 | Virulent pathogen | Pathogen capable of causing severe disease (strongly pathogenic). |
| 2 | Avirulent pathogen | Pathogen which is unable to cause disease (non-pathogenic). |
| 3 | Host plant | Plant species on which pathogen harbors and gets nourishment and shelter. |
| 4 | Nonhost pathogen | Pathogen for which particular plant species is not a host. |
| 5 | Nonhost plant | Plant species on which pathogen fails to colonize. |
| 6 | Pathogenicity | Ability of pathogen to cause disease. |
| 7 | Virulence | Degree of pathogenicity as indicated by the severity of disease produced and the ability to invade the host |
| 8 | Compatible interaction (Susceptible interaction) | Plant-pathogen interaction which favors a pathogen to harbor on plant and makes plant susceptible to pathogen. |
| 9 | Incompatible interaction (Resistant interaction) | Plant-pathogen interaction in which a pathogen harboring on plant no longer grows due to resistance reaction by plant. |
| 10 | Hypersensitive response (HR) | Reaction by a plant to an invading pathogen in which the plant tissue around infected sites dies (cell death) in order to prevent further spread of the infection. |
| 11 | Programmed cell death (PCD) | Sequence of events resulting in controlled and organized destruction of cell. |
| 12 | PAMPs | Pathogen-associated molecular patterns, molecules associated with group of pathogen. For example bacterial flagellin. |
| 13 | PRR | Plant pathogen- or pattern recognition receptor, Receptor present on plasma-membrane of cell which recognizes PAMPs. |
| 14 | Plant Immunity | Plant immunity is the inherent or induced capacity of plants to withstand or ward off biological attack by pathogens. |
| 15 | PTI (PAMP/Pathogen/Pattern Triggered Immunity) | Immunity which is triggered in plants from pathogen perception due to recognition of PAMP by PRR. |
| 16 | Effector | Proteins (ligand) secreted by bacteria, oomycetes, fungi and nematodes that modulate plant innate immunity and enable parasitic infection. |
| 17 | R-protein | Proteins which directly or indirectly recognize effectors and mediate defense response. |
| 18 | Effector Triggered Immunity (ETI) | Immunity which is triggered by pathogen perception due to recognition of effectors by resistance protein (R-protein) in cell. |
| 19 | Avirulent factor | Effectors of pathogen for which plant contains recognizing resistance protein (R-protein). |
| 20 | Disease susceptibility genes | Host plant gene which can be manipulated by pathogen and makes plant susceptible to pathogen. |
| 21 | *R*-gene mediated resistance | Resistance mechanism elicited in plants due to recognition of avirulent factor by R proteins. |
| 22 | Nonhost Resistance | Broad spectrum plant defense which provide immunity to all members of a plant species against all isolates of pathogen which are pathogenic to other plant species. |
|  | | |

Source:

<http://bugs.bio.usyd.edu.au/learning/resources/PlantPathology>

Jones, J. D., and Dangl, J. L. (2006). The plant immune system. *Nature*, *444*, 323-329.

Lockshin, R. A., and Zakeri, Z. (2004). Apoptosis, autophagy, and more. *Int J Biochem Cell Biol*, *36*, 2405-2419.

Senthil-Kumar, M., & Mysore, K. S. (2013). Nonhost resistance against bacterial pathogens: retrospectives and prospects. *Annu. Rev. Phytopathol*., 51, 407-427.

Shaner, G., Stromberg, E. L., Lacy, G. H., Barker, K. R., and Pirone, T. P. (1992). Nomenclature and concepts of pathogenicity and virulence. *Annu. Rev. Phytopathol.*, *30*, 47-66.
