# Supplementary material for: Role of proline and pyrroline-5-carboxylate metabolism in plant defense against invading pathogens
Source: Front Plant Sci. 2015 Jul 6;6:503. doi: 10.3389/fpls.2015.00503 (PMC4491715; doi:10.3389/fpls.2015.00503)
Supplement: Supplementary file 1 [file Presentation_1.ZIP › presentation 1/Senthil-Kumar_SupplementaryMaterial/Supplementary table S2.DOCX]

**Supplementary table S2. List of *Arabidopsis thaliana* genes proposed to be involved in proline-P5C metabolism and their role in plant defense against pathogen and abiotic stress tolerance.**

| **Sl. No.** | **Gene name** | **Gene ID** | **Biological role** | **Reference** |
| --- | --- | --- | --- | --- |
| 1 | *Pyrroline,5-carboxylate synthase 1 (P5CS1)* | *At2g39800* | It catalyzes first rate limiting step of proline biosynthesis. Transcripts levels increase under, drought and salt stress. It’s increase is attributed for high levels of proline in the cell. | Hu et al.,1992  Yoshiba et al.,1995  Yoshiba et al.,1997  Szekely et al., 2008 |
| 2 | *Pyrroline,5-carboxylate synthase 2 (P5CS2)* | *At3g55610* | It catalyzes first rate limiting step of proline biosynthesis. Transcript level increases during pathogen infection. This gene is suggested to play role in plant-pathogen interaction. | Hu et al.,1992  Yoshiba et al.,1995  Yoshiba et al.,1997  Fabro et al., 2004 |
| 3 | *Pyrroline,5-carboxylate reductase (P5CR)* | *At5g14800* | It catalyzes second step of proline biosynthesis. Transcript level increases under drought stress and during recovery. | Miller et al., 2009  Szoke et al., 1992  Verbruggen et al., 1993 |
| 4 | *Proline dehydrogenase 1 (ProDH1)* | *At3g30775* | It catabolizes proline in mitochondria. Transcription of gene is downregulated under drought and salinity. Transcript level is upregulated during pathogen infection. It is implicated in nonhost resistance and resistance to avirulent pathogen. | Kiyosue et al., 1996  Verbruggen et al., 1996  Verslues et al., 2007  Sharma and Verslues, 2010  Cecchini et al., 2011  Senthil-Kumar and Mysore, 2012 |
| 5 | *Proline dehydrogenase 2 (ProDH2)* | *At5g38710* | It catabolizes proline in mitochondria. Transcript level is upregulated during drought stress recovery. | Funck et al., 2010  Sharma and Verslues, 2010  Cecchini et al., 2011  Senthil-Kumar and Mysore, 2012 |
| 6 | *Pyrroline,5-carboxylate dehydrogenase (P5CDH)* | *At5g62530* | It catabolizes P5C in mitochondria. Transcript level is up regulated under virulent pathogen infection. | Szoke et al., 1992  Deuschle et al., 2001  Deuschle et al., 2004  Borsani et al., 2005 |
| 7 | *Similar to RCD-5 (SRO5)* | *At5g62520* | Probable inactive ADP-ribosyltransferase. It is expressed under salt stress and provides resistance. Antisense gene pair of *P5CDH* and *SRO5* generates natural siRNAs and regulates *P5CDH* mRNA levels in the cell under salt stress. | Borsani et al., 2005  Babajani et al., 2009 |
| 8 | *Ornithine δ-aminotransferase (δ-OAT)* | *At5g46180* | It catalyzes reversible transamination of P5C to ornithine. It is implicated for role in nonhost resistance. Transcript of gene is upregulated under salt stress. | Delauney et al., 1993  Roosens et al., 1998  Senthil-Kumar and Mysore, 2012 |
| 9 | *Proline transporter 1 (ProT1)* | *At2g39890* | Proline transporter localized at plasma membrane. Transcript expression is high in stem and flowers than other parts of plant. | Rentsch et al., 1996  Grallath et al., 2005 |
| 10 | *Proline transporter 2 (ProT2)* | *At3g55740* | Proline transporter localized at plasma membrane. Expression is high in roots. It is induced under salt stress. | Rentsch et al., 1996  Grallath et al., 2005 |
| 11 | *Proline transporter 3 (ProT3)* | *At2g35590* | Proline transporter localized at plasma membrane. | Rentsch et al., 1996  Grallath et al., 2005 |
| 12 | *Basic aminoacid carrier 1 (BAC1)* | *At2g33820* | Basic aminoacid transporter localized at mitochondrial membrane. It exports ornithine and arginine from mitochondria to cytosol. | Hoyos et al., 2003 |
| 13 | *Basic aminoacid carrier 2 (BAC2)* | *At1g79900* | Mitochondria localized transporter exports ornithine and arginine from mitochondria. It is induced by ABA or osmotic stress treatments. | Hoyos et al., 2003  Palmieri et al., 2006 |

*highlighted rows indicate genes implicated in plant-pathogen interaction

**References**

Babajani, G., Effendy, J. and Plant, A.L. (2009). Sl-SROl1 increases salt tolerance and is a member of the radical-induced cell death 1-similar to RCD1 gene family of tomato. *Plant Sci*. 176, 214-222.

Borsani, O., Zhu, J., Verslues, P.E., Sunkar, R., and Zhu, J.K. (2005). Endogenous siRNAs derived from a pair of natural cis-antisense transcripts regulate salt tolerance in Arabidopsis. *Cell*.123, 1279-1291.

Cecchini, N. M., Monteoliva, M. I., and Alvarez, M. E. (2011). Proline dehydrogenase contributes to pathogen defense in Arabidopsis. *Plant Physiol*. 155, 1947-1959.

Delauney, A., Hu, C., Kishor, K., and Verma, D. (1993). Cloning of ornithine d-aminotransferase cDNA by trans-complementation in *Escherichia coli* and regulation of proline biosynthesis. *J Biol Chem*. 268, 18673-18678.

Deuschle, K., Funck, D., Forlani, G., Stransky, H., Biehl, A., Leister, D., et al. (2004). The role of Δ1-pyrroline-5-carboxylate dehydrogenase in proline degradation. *Plant Cell*. 16, 3413-3425.

Deuschle, K., Funck, D., Hellmann, H., Daschner, K., Binder, S., and Frommer, W.B. (2001). A nuclear gene encoding mitochondrial delta1-pyrroline-5-carboxylate dehydrogenase and its potential role in protection from proline toxicity. *Plant J*. 27, 345-355.

Fabro, G., Kovacs, I., Pavet, V., Szabados, L., Alvarez, M.E. (2004). Proline accumulation and *AtP5CS2* gene activation are induced by plant-pathogen incompatible interactions in Arabidopsis. *Mol Plant Microbe Interact*. 17, 343-350.

Funck, D., Eckard, S., and Muller, G. (2010). Non-redundant functions of two proline dehydrogenase isoforms in Arabidopsis. *BMC Plant Biol*. 10, 70.

Grallath, S., Weimar, T., Meyer, A., Gumy, C., Suter-Grotemeyer, M., Neuhaus, J. M., and Rentsch, D. (2005). The AtProT family. Compatible solute transporters with similar substrate specificity but differential expression patterns. *Plant Physiol*. 137, 117-126.

Hoyos, M.E., Palmieri, L., Wertin, T., Arrigoni, R., Polacco, J.C., and Palmieri, F. (2003). Identification of a mitochondrial transporter for basic amino acids in *Arabidopsis thaliana* by functional reconstitution into liposomes and complementation in yeast. *Plant J*. 33, 1027-1035.

Hu, C.A., Delauney, A.J., and Verma, D.P.S. (1992). A bifunctional enzyme (delta1-pyrroline-5-carboxylate synthase) catalyzes the first two steps in proline biosynthesis in plants. *Proc Natl Acad Sci*. 89, 9354-9358.

Kiyosue, T., Yoshiba, Y. Yamaguchi-Shinozaki, K., and Shinozaki, K. (1996). A nuclear gene encoding mitochondrial proline dehydrogenase, an enzyme involved in proline metabolism, is upregulated by proline but downregulated by dehydration in Arabidopsis. *Plant Cell*. 8, 1323-1335.

Miller, G., Honig, A., Stein, H., Suzuki, N., Mitler, R., and Zilberstein, A. (2009). Unraveling delta1-pyrroline-5-carboxylate-proline cycle in plants by uncoupled expression of proline oxidation enzymes. *J Biol Chem*. 289, 26482-26492.

Palmieri, L., Todd, C. D., Arrigoni, R., Hoyos, M. E., Santoro, A., Polacco, J. C., and Palmieri, F. (2006). Arabidopsis mitochondria have two basic amino acid transporters with partially overlapping specificities and differential expression in seedling development. *Biochim Biophys* *Acta* –*Bioenergetics*. 1757, 1277-1283.

Rentsch, D., Hirner, B., Schmelzer, E., and Frommer, W. B. (1996). Salt stress-induced proline transporters and salt stress-repressed broad specificity amino acid permeases identified by suppression of a yeast amino acid permease-targeting mutant. *Plant Cell.* 8, 1437-1446.

Roosens, N. H., Thu, T. T., Iskandar, H. M., and Jacobs, M. (1998). Isolation of the ornithine-delta-aminotransferase cDNA and effect of salt stress on its expression in *Arabidopsis thaliana*. *Plant Physiol*. 117, 263-271.

Senthil‐Kumar, M., and Mysore, K. S. (2012). Ornithine‐delta‐aminotransferase and proline dehydrogenase genes play a role in non‐host disease resistance by regulating pyrroline‐5‐carboxylate metabolism‐induced hypersensitive response. *Plant Cell Environ*. 35, 1329-1343.

Sharma, S., and Verslues, P.E. (2010). Mechanisms independent of ABA or proline feedback have a predominant role in transcriptional regulation of proline metabolism during low water potential and stress recovery. *Plant Cell Environ*. 33, 1838-1851.

Szekely, G., Abraham, E., Cselo, A., Rigo, G., Zsigmond, L., Csiszar, J. et al. (2008). Duplicated P5CS genes of Arabidopsis play distinct roles in stress regulation and developmental control of proline biosynthesis. *Plant J*. 53, 11-28.

Szoke, A, Miao, G. H., Hong, Z. L., and Verma, D. P. S. (1992). Sub cellular location and delta-pyrroline-5-carboxylate reductase in root nodule and leaf of soybean. *Plant Physiol*. 99, 1642-1649.

Verbruggen, N., Hua, X.J., May, M., and VanMontagu, M. (1996). Environmental and developmental signals modulate proline homeostasis: Evidence for a negative transcriptional regulator. *Proc Natl Acad Sci*. 93, 8787-8791.

Verbruggen, N., Villarroel, R., and Vanmontagu, M. (1993). Osmoregulation of a pyrroline-5-carboxylate reductase gene in *Arabidopsis thaliana*. *Plant Physiol*. 103, 771-781.

Verslues, P.E., Kim, Y.S., and Zhu, J.K. (2007). Altered ABA, proline and hydrogen peroxide in an Arabidopsis glutamate: glyoxylate aminotransferase mutant. *Plant Mol Biol*. 64, 205-217.

Yoshiba, Y., Kiyosue, T., Katagiri, T., Ueda, H., Mizoguchi, T., Yamaguchi‐Shinozaki, K., et al. (1995). Correlation between the induction of a gene for Δ1‐pyrroline‐5‐carboxylate synthetase and the accumulation of proline in *Arabidopsis thaliana* under osmotic stress. *Plant J.* 7, 751-760.

Yoshiba, Y., Kiyosue, T., Nakashima, K., Yamaguchi-Shinozaki, K., and Shinozaki, K. (1997). Regulation of levels of proline as an osmolyte in plants under water stress. *Plant and Cell Physiol*, 38, 1095-1102.
